# A136 MÉNÉTRIER’S DISEASE-LIKE PRESENTATION IN A PEDIATRIC PATIENT AS A FIRST PRESENTATION OF SYSTEMIC LUPUS ERYTHEMATOSUS

**DOI:** 10.1093/jcag/gwae059.136

**Published:** 2025-02-10

**Authors:** M Hussain, Z Alsaffar, N Ahmed

**Affiliations:** Pediatrics, McGill University, Montréal, QC, Canada; Pediatrics, McGill University, Montréal, QC, Canada; Pediatrics, McGill University, Montréal, QC, Canada

## Abstract

**Background:**

Ménétrier’s disease (MD) is an uncommon gastric disorder characterized by hypertrophic gastric mucosal folds, excess mucus secretion, and protein-losing gastropathy. It is mainly seen in adult patients, with around 100 pediatric cases reported to date, often associated with cytomegalovirus (CMV) infection. Systemic lupus erythematosus (SLE) is a autoimmune condition that can affect multiple systems. While MD and SLE are separately rare, there have been reports of MD preceding SLE in adult patients. However, no cases have documented MD as the initial presentation leading to a diagnosis of SLE in pediatric patients.

**Aims:**

This case report aims to describe the first documented instance of a pediatric patient presenting with suspected Ménétrier’s disease, whose clinical course led to a diagnosis of systemic lupus erythematosus.

**Methods: Case Summary:**

A 13-year-old boy with a history of autism spectrum disorder, presented with anasarca and multiple systemic symptoms, including musculoskeletal pain, fatigue, mouth ulcers, non-productive cough, and generalized edema. On admission, he was hypotensive with mild conjunctival injection, peri-orbital edema, ascites, and non-pitting edema in the lower limbs. Lab tests showed leukopenia, neutropenia, thrombocytopenia, elevated ferritin, and hypoalbuminemia. Imaging revealed bilateral pleural effusions and ascites, along with thickened gastric folds on CT scan raising suspicion for Ménétrier’s disease. An upper GI endoscopy showed atypical thick folds with scaliness in the stomach, but the biopsy was negative for H. pylori, CMV, showed no significant inflammation, no foveolar metaplasia, no viral cytopathic changes and had well-preserved morphology in esophageal, gastric and duodenal biopsies. A kidney biopsy was done in the context of proteinuria and confirmed lupus nephritis.

**Results:**

He was diagnosed with systemic lupus erythematosus (SLE) and treated with steroids, Plaquenil, and Mycophenolate Mofetil. Post-treatment, his symptoms, including ascites and edema, resolved, and his lab values, such as blood counts, albumin, and C3/C4 levels, normalized. The patient showed significant clinical improvement following SLE-targeted therapy.

**Conclusions:**

This case highlights the complexity and diagnostic challenges in differentiating Ménétrier’s disease from systemic lupus erythematosus, particularly when both disorders present with overlapping features. In addition, it suggests that in rare instances, MD may be a manifestation of a broader systemic inflammatory process such as SLE. Multidisciplinary approach and comprehensive evaluation are essential for accurate diagnosis and management in such complex cases.

**Funding Agencies:**

None

